# Lymphome non Hodgkinien intramusculaire primitif chez le sujet jeune: à propos d’un cas et revue de la littérature

**DOI:** 10.11604/pamj.2016.25.223.10600

**Published:** 2016-12-07

**Authors:** Soufya Majdoul, Nabil Omari, Youness Allali, Reda Ghabri, Nadia Benchakroun, Mostafa Fadili, Nezha Tawfiq, Hassan Jouhadi, Souha Sahraoui, Mohamed Nechad, Abdelatif Benider

**Affiliations:** 1 Centre Mohammed VI pour le Traitement des Cancers, CHU Ibn Rochd Casablanca, Maroc; 2 Service de Traumatologie et d’Orthopédie Aile 4, CHU Ibn Rochd Casablanca, Maroc; 3 Faculté de Médecine et Pharmacie de Casablanca, Maroc

**Keywords:** Lymphome non hodgkinien, muscle, primitif, traitement, Non-Hodgkin's lymphoma, muscle, primitive, treatment

## Abstract

Le lymphome non hodgkinien (LNHK) extra-ganglionnaire primitif est rare et sa localisation intramusculaire primitive est exceptionnelle puisqu'elle est observée chez moins de 0,5% des patients. Ils touchent généralement des hommes dont l'âge moyen est de 70 ans. Le traitement standard associe l'exérèse chirurgicale, la chimiothérapie et la radiothérapie. Nous rapportons un cas d'un jeune patient de 31 ans qui s'est présenté pour un syndrome pseudotumorale musculaire au niveau de la jambe droite dont l'examen anatomopathologique avait conclu un lymphome malin non hodgkinien à grandes cellules B intramusculaire. Le patient a été traité par chimiothérapie exclusive avec une rémission complète.

## Abstract (en)

Primary extra-ganglionic non-Hodgkin's lymphoma (NHL) is rare and its primary intramuscular location is exceptional, as it account for less than 0.5% of all patients. It generally affects men, with an average age of 70 years. Standard treatment is based on surgical excision combined with chemotherapy and radiation therapy. We report the case of a 31-year old patient presenting with muscular pseudotumoral syndrome at the level of the right leg. Histological examination showed intramuscular large B-cell non-Hodgkin's lymphoma. The patient underwent exclusive chemotherapy with complete remission.

## Introduction

Les localisations des lymphomes malins aux muscles striés sont exceptionnelles. Ils sont observés chez moins de 1,5% des cas [1, 2]. Les signes d´appel clinique ne sont pas spécifiques. L´IRM est l´examen de choix. La biopsie musculaire fait le diagnostic de certitude. Le traitement est basé sur la chimiothérapie et radiothérapie. Le traitement standard associe l'exérèse chirurgicale de la tumeur et une chimiothérapie, suivie parfois d'une radiothérapie [3]. Nous rapportons l'observation d'un jeune patient présentant un lymphome malin non hodgkinien à grandes cellules B diffus du muscle squelettique dont le traitement était médical par chimiothérapie seule avec une rémission complète.

## Patient et observation

**Figure 1:**
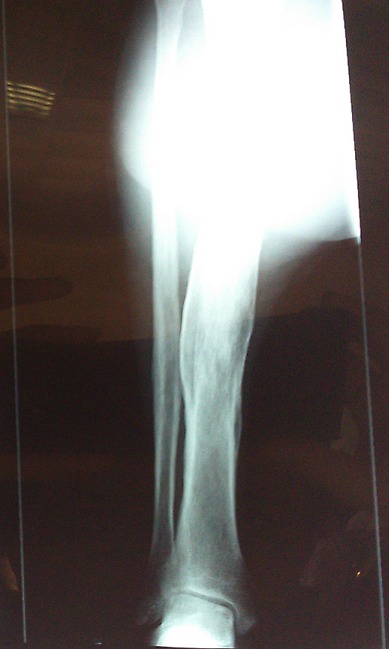
Radiographie standard du membre inférieur: une infiltration des parties molles

**Figure 2:**
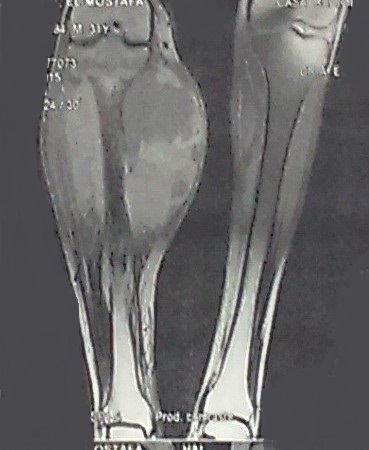
IRM de la jambe droite: coupe sagittale en T2 montrant une masse des deux tiers supérieurs de jambe droite mesurant 19x13 cm prenant le contraste de façon hétérogène avec composantes liquidiennes nécrotiques

**Figure 3:**
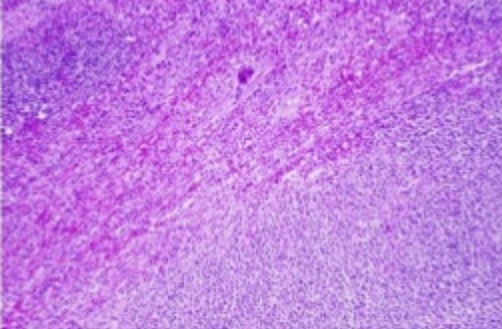
Expression de CD 20 par les cellules

Un patient M.J, âgé de 31 ans, sans antécédents pathologiques particuliers, présentait une tuméfaction au niveau de la face postérieure de la jambe droite augmentant progressivement de volume apparue 8 mois avant son admission, sans notion de traumatisme, évoluant dans un contexte d'apyrexie et de conservation de l'état général. L'examen clinique, retrouvait une masse ulcéro-bourgeonnante de 19x13x5 cm, douloureuse à la palpation, adhérente au plan musculaire profond, localisée au niveau de la face postérieure des deux tiers supérieurs de la jambe droite, l'examen aires ganglionnaire était normal. Les radiographies de la jambe et du genou ont montré une infiltration des parties molles sans atteinte osseuse (Figure 1). L'IRM de la jambe droite montrait un processus lésionnel d'allure tumorale intéressant les loges musculaires en regard des deux tiers supérieurs de jambe droite mesurant 19x13 cm prenant le contraste de façon hétérogène avec composante s liquidiennes nécrotiques, il est en hyposignal en T1 et discret hypersignal en T2 FAT SAT et surtout hypersignal hétérogène T2 STIR (Figure 2). Le bilan d'extension fait de TDM thoraco-abdolmino-pelvienne était normal. Les sérologies virales étaient négatives. Une biopsie a été réalisée. L'étude anatomopathologique a révélé une prolifération tumorale cellulaire, dense et diffuse avec une nécrose tumorale importante et un index mitotique élevé. L'étude immunohistochimique a montré une expression diffuse et intense de CD 20 ce qui a permis le diagnostic positif d'un lymphome B diffus a grandes cellules (Figure 3). Après une discussion e réunion de concertation multidisciplinaire, le patient a reçu six cycles de chimiothérapie type R-CHOP (rituximab, cyclophosphamide, doxorubicine (Adriamycin), vincristine (Oncovin^®^), et prednisone) avec une rémission complète après 24 mois de recul.

## Discussion

Les localisations primitives musculaires des lymphomes malins non hodgkiniens sont exceptionnelles, représentant 1,5% des lymphomes non hodgkiniens extraganglionnaires [1, 2]. L'atteinte musculaire du lymphome peut être liée à trois mécanismes : envahissement du muscle à partir d'une adénopathie voisine, dissémination métastatique ou lymphome primitif du muscle squelettique [4, 5]. Dans une grande étude rétrospective de la Mayo Clinic, ils ont rapporté une incidence de lymphome primaire du muscle de 8 de 7000 cas de lymphomes malins seulement. Le lymphome primitif du muscle est de mauvais pronostic [6]. Les lymphomes musculaires primitifs surviennent principalement chez les hommes, d'âge moyen 68 ans [2, 6–8]. La localisation chez le sujet jeune rapporté dans notre observation est exceptionnelle.

Le lymphome non hodgkinien atteint 60 fois plus les patients immunodéprimés (par exemple les patients infectés par le virus de l´immunodéficience humaine, les patients âgés, les patients sans antécédents de traitement antirétroviral actif, et les patients avec numération des CD4 inférieur à 100 cellules / mL) que chez les patients normaux, dans notre cas le patient avait des sérologies négatives [3]. Les signes d'appel clinique ne sont pas spécifiques. Leur découverte par les patients est liée, le plus souvent, à la perception d'une tuméfaction. Parfois, la notion d'un rapport avec une blessure est mentionnée dans l'histoire de la maladie [2, 7, 8]. Ces tumeurs se développent aux dépens d'un compartiment musculaire, puis se propagent à d'autres compartiments contigus [7]. Elles peuvent être responsables d'un syndrome des loges ou même d'une rhabdomyolyse menaçant le pronostic vital en raison de désordres hydroélectrolytiques majeurs [2]. Ces lymphomes sont préférentiellement localisés aux membres inférieurs 88% des cas, puis à l'abdomen [8], les autres atteintes sont moins habituelles et notamment aux membres supérieurs et au pelvis. L'IRM est plus sensible que l'examen tomodensitométrique pour dépister les localisations musculaires des lymphomes et établir sa relation aux éléments vasculaires et nerveux [7]. Il permet d'orienter le diagnostic devant la présence de certains signes en faveur de l'atteinte lymphomateuse tels que l'atteinte multi-compartimentale et longitudinale le long des fibres musculaires, le respect de quelques septa graisseux intramusculaires, la visualisation de structures vasculaires au sein de la masse, ainsi que l'atteinte possible du tissu sous-cutané et la prise de contraste marginale des septa [7, 9, 10].

Des études récentes ont aussi suggéré que le PET-scan pourrait être une technique prometteuse dans cette indication; elles ont relevé un hypermétabolisme au niveau musculaire chez ces patients [10]. Le diagnostic de certitude repose sur l'examen histologique d'une biopsie chirurgicale de la tumeur. Dans la grande majorité des cas, la masse est un lymphome de type B [11]. (Plus de 95%des cas), dont le pronostic semble-t-il meilleur que celui des lymphomes de type T [5]. La décision du type de traitement devrait toujours résulter de discussions multidisciplinaires (oncologues, radiologues, anatomopathologistes, chirurgiens orthopédiques). La prise en charge ne devrait pas être exclusivement chirurgicale car elle peut être responsable de mutilations [1, 10]. Elle peut ne pas être efficace et entraîner des récidives locales et une dissémination à distance. La chimiothérapie est le traitement de choix. L'association d'un anticorps monoclonal anti-CD20 et d'une chimiothérapie de type CHOP reste le traitement de choix. La radiothérapie n'est pas indiquée en cas de réponse complète au traitement de première ligne [12].

## Conclusion

Le lymphome musculaire est rare. Seule la biopsie qui permet le diagnostic de certitude, la chirurgie peut être évité, vu le lymphome est une tumeur très chimio sensible, la radiothérapie n'est pas indiquée qu'en cas de maladie résiduelle.
